# Vitamin C supplementation modulates gene expression in peripheral blood mononuclear cells specifically upon an inflammatory stimulus: a pilot study in healthy subjects

**DOI:** 10.1007/s12263-014-0390-x

**Published:** 2014-03-07

**Authors:** Raffaella Canali, Lucia Natarelli, Guido Leoni, Elena Azzini, Raffaella Comitato, Oezgur Sancak, Luca Barella, Fabio Virgili

**Affiliations:** 1 Food and Nutrition Research Centre, Consiglio per la Ricerca e Sperimentazione in Agricoltura, Via Ardeatina 546, 00178 Rome, Italy; 2 Bayer Consumer Care Ltd, Basel, Switzerland

**Keywords:** Cytokines, Gene expression, Inflammation, PBMNC, Vitamin C

## Abstract

In order to study the effects of vitamin C supplementation on gene expression and compare its action between physiological and inflammatory conditions, a pilot study was set up utilizing microarray and qPCR technologies. Five healthy volunteers were supplemented with 1 g vitamin C (Redoxon^®^) per day for five consecutive days. Peripheral blood mononuclear cells (PBMNC) were isolated before and just after the last supplementation, and RNA was isolated for the Affymetrix gene 1.0 ST chip analysis. PBMNC were also, ex vivo, treated with LPS, and gene expression was quantified by means of a “Human NFkB Signaling” qPCR array. Only a very moderate effect on the baseline gene expression modulation was associated with vitamin C supplementation. However, in spite of the limited number of subjects analyzed, vitamin C supplementation resulted in a markedly different modulation of gene expression upon the inflammatory stimulus, specifically at the level of the MyD88-dependent pathway and of the anti-inflammatory cytokine IL-10 synthesis. This study suggests that vitamin C supplementation in healthy subjects, not selected according to a specific genetic profile, consuming an adequate amount of vitamin C, and having a satisfactory vitamin C plasma concentration at the baseline, does not result in a significant modification of gene expression profile. Under this satisfactory micronutrient status, supplementation of vitamin C is “buffered” within a homeostatic physiological equilibrium. Differently, following a second “hit” constituted of an inflammatory stimulus such as LPS, able to trigger a critical burst to the normal physiological state, the higher availability of ascorbic acid emerges, and results in a significant modulation of cell response.

## Introduction

Vitamin C or ascorbic acid (AA) is essential for life in humans as the capacity to synthesize it has been lost in course of our evolution. The physiological functions of AA are largely dependent on the oxidoreductive properties of this vitamin. Ascorbic acid (AA) acts as a cofactor in Cu and Fe hydroxylase, and monooxygenase enzymes involved in the synthesis of collagen, carnitine, coagulation factor V, and neurotransmitters. Several studies have demonstrated that AA status is related to a decreased risk of a wide range of pathologies, such as stroke (Gale et al. 1995) or cardiovascular disease (Salonen et al. 2003). In this context, AA supplementation has been reported to ameliorate symptoms and to enhance the expression of specific immune response markers in clinical conditions (Wintergerst et al. 2006). It is interesting to mention that most of the available studies addressing the effects of AA on human health considered the supplementation of this vitamin together with other nutrients (usually zinc or within a multivitamin–multimineral formula), while a real understanding of its mechanism of action would possibly require the supplementation as single components (Lykkesfeldt and Poulsen 2010).

A role of vitamin C as an antioxidant molecule has also been recognized, largely based on in vitro studies. At present, available data supporting the role of ascorbic acid as an in vivo “reductant” are conflicting (Traber and Stevens 2011). According to this hypothesis, vitamin C supplementation has been reported to decrease the steady-state level of oxidative DNA and lipid damage, in mononuclear blood cells of smoker and in guinea pigs (Moller et al. 2004; Chen et al. 2000); on the other hand, the administration of 1,000 mg of ascorbic acid plus alpha-tocopherol had no effects on oxidative stress and DNA damage in aged healthy adults (Retana-Ugalde et al. 2008). In general, acting as an “antioxidant,” AA would contribute in maintaining cellular and extracellular redox balance, thereby protecting against reactive oxygen species generated during cellular events characterized by an increased production of oxidant species, such as in the course of respiratory burst and inflammatory response.

However, more recently, the concept of “antioxidant molecules” has been reconsidered and their biological functions are no longer considered to be simply due to their ability to act as “electron donors;” rather, they appear to act by modulating signaling and gene expression. In vivo and in vitro studies have shown that some of the effects associated with AA are at transcriptional level (Park et al. 2009; Shin et al. 2004; Belin et al. 2009). Despite a number of solid indications, the mechanism by which ascorbic acid affects gene expression is still unclear.

In order to gain a better understanding of the effects of vitamin C supplementation on gene expression in both physiological “homeostatic” conditions and in the presence of a pro-inflammatory stimulus, we performed a pilot explorative intervention study based on the utilization of a microarray approach and also designed to establish an “in vivo–ex vivo” structure.

## Materials and methods

### Study design

Five healthy subjects (three males and two females, aged 25–40) were recruited for a short-term (5 days) intervention study, in order to assess the effect of vitamin C on gene expression modulation.

Subjects were recruited among the employees of the Institute and provided written informed consent to participate in the study. All participants were in good health as determined by a medical history questionnaire and clinical laboratory tests. All subjects fulfilled the following eligibility criteria, they are as follows: (1) no history of chronic disease, (2) no antibiotic or supplemental vitamin or mineral use for 4 weeks before the beginning of the study, (3) nonsmoking, (4) low physical activity, and (5) no drugs or nonsteroidal inflammatory drugs (NSAID) for at least 10 days before and during the supplementation period. The study protocol was approved by the internal ethical committee and was in accordance with the Declaration of Helsinki 1975, as revised in 1983. Subjects were instructed to maintain their usual habits (diet and physical activity) during the 5 days of Vitamin C supplementation. The subjects were asked to abstain from eating vegetables, fruits, and drinking alcohol the evening before the day of blood withdrawals. Subjects were asked to assume one tablet of Redoxon^®^ (Bayer) a day (containing 1 g of ascorbate), for 5 days. A baseline blood withdrawal was performed in fasting condition, just before the first vitamin C tablet. Tablets were provided to the volunteers, every morning at the same hour for the following 4 days of the study. The last tablet was supplemented about 1, 5 h before the second and last blood withdrawal, performed again in fasting condition. The duration of the supplementation and the time of the blood withdrawal after the last Redoxon^®^ tablet were planned on the basis of previously published study (Jeng et al. 1996; Moller et al. 2004) in order to have a combination of a steady-state concentration and acute effects of circulating vitamin C.

Plasma and peripheral blood mononuclear cells (PBMNC) were obtained as described below. PBMNC from each subject were splitted into three aliquots: one was stored in lysis buffer at −80 °C and utilized to isolate RNA for the Affymetrix gene array analysis. The other two aliquots were resuspended in RPMI 1,640 w/o phenol red (plus pen/strep and glutamine) containing 10 % of autologous plasma: one was incubated with 10 μg/ml lipopolysaccharides (LPS) (Sigma-Aldrich) for 5 h at 37 °C. At the end of the incubation time, the medium was collected and stored at −80 °C for the assessment of cytokine release in response to the inflammatory stimuli. RNA was extracted from the pelleted cells for gene expression analysis using the Human NFκB Signaling 96 StellARRray™ qPCR array. Both microarray and the qPCR NFkappaB 96 gene card analysis have been performed according to the MIAME guidelines and deposited in NCBI Gene Expression Omnibus and are accessible through GEO series accession number GSE54477; moreover, data will be available in the Nutritional Phenotype Database (dbNP) (van Ommen et al. 2010). Data obtained with PBMNC isolated after vitamin C supplementation (supplemented PBMNC) were compared to those obtained with PBMNC isolated before the supplementation (baseline PBMNC), for each subject.

### Plasma vitamin C

Immediately after blood withdrawal, an aliquot of plasma was diluted with an equal amount of 10 % metaphosphoric acid with 2 mM EDTA to prevent the vitamin C oxidation and stored at −80 °C for further uses. Total ascorbic acid (ascorbate and dehydroascorbic acid (DHA)) was extracted according to the method described by Margolis et al. (1997). Quantitative analysis was performed using an HPLC system coupled with a coulometric detector (ESA model 580; Chelmsford, MA, USA).

### PBMNC isolation and treatment

PBMNC were isolated from blood by centrifugation in Ficoll gradient density (Ficoll-Paque PLUS, GE Healthcare). Briefly, venous blood was obtained from the healthy volunteers and heparin was used as anticoagulant. Blood was centrifuged (1,500×*g* for 15 min) and plasma separated from RBC and WBC. RBC and WBC were suspended with RPMI 1640 w/o phenol red (Euroclone) (plus pen/strep, glutamine and w/o serum) and PBMNC isolated by centrifugation in Ficoll gradient density. After centrifugation (400×*g* for 30 min at 20 °C), PBMNC-rich pellet was recovered and washed in PBS. PBMNC preparation was checked for cell purity and total count.

### Microarray analysis

RNA extraction from PBMNC isolated from each subjects before and after the vitamin C supplementation was performed using RNAeasy^®^ Plus Mini kit (Qiagen) according to the manufacturer’s protocol. Concentration, purity, and integrity were assessed using the Agilent 2100 bioanalyzer (Agilent Technologies), before gene expression analysis. About 2 μg of each isolated RNA samples was sent to the Institute of Molecular Oncology Foundation (IFOM, Milano), to be processed and hybridized on Gene 1.0 ST array chips according to Affymetrix protocol. These arrays allow the assessment of the expression level of 28,869 genes represented by approximately 26 probes spread across the full length of the gene, providing a complete and accurate picture of gene expression than the classical 3′-based expression array designs. Cell intensity files for each Gene 1.0 ST chip processed were generated utilizing Command Console software. The lists of significantly modulated genes were analyzed by using a DAVID Web server in order to identify statistically overrepresented biological processes annotated in Gene Ontology (GO) (Huang et al. 2009). Enriched GO biological processes (level 5) were identified according to *p* value threshold <0.05 and clusterized utilizing the “Functional Annotation Clustering” tool included in DAVID. Representative pathway categories from the most statistically significant clusters were manually selected and listed as they appear in Table 1.

**Table 1:** Effect of vitamin C supplementation on gene expression level in PBMNC, assessed by means of Affymetrix and qPCR platforms

Official gene symbol	Gene name	Affymetrix		Representative pathways^c^	QPCR	
		FC^a^	*p* value^b^		FC^d^	*p* value^e^
CANX	Calnexin	1.13	0.0014	Chromatin organization		
CANX(1)	Calnexin1			Chromatin organization	0.18 ± 0.12	N.S.^f^
CANX(2)	Calnexin2			Chromatin organization	0.53 ± 0.37	0.021
ARF3	ADP-ribosylation factor 3	0.98	0.0003	Chromatin organization		
SMNDC1	Survival motor neuron domain containing 1	0.95	0.0054	Chromatin organization		
RPS24	Ribosomal proteinS24	0.89	0.0015	Chromatin organization		
MYST2	Histone acetyltransferase MYST2	0.87	0.0320	Chromatin organization; ribonucleoprotein complex biosynthesis		
NONO	Non-POU domain containing	0.81	0.0040	Ribonucleoprotein complex biosynthesis; RNA processing		
ZNF259	Zinc finger protein 259	0.81	0.0017	Ribonucleoprotein complex biosynthesis; RNA processing		
FNTA	Farnesyl-protein transferase alpha subunit	0.79	0.0041	Ribonucleoprotein complex biosynthesis; translation; RNA processing		
Y_RNA	Small noncoding RNA components of the Ro ribonucleoprotein	0.77	0.0101	Ribonucleoprotein complex biosynthesis; translation; RNA processing		
HRH4	Histamine receptor H4	0.74	0.0191	Ribonucleoprotein complex biosynthesis; translation; RNA processing		
IK	IK cytokine	0.73	0.0234	Ribonucleoprotein complex synthesis	0.15 ± 0.44	N.S.
CTLA4	Cytotoxic T-lymphocyte antigen 4	0.7	0.0234	RNA processing		
U1		0.68	0.0045	RNA processing		
5S_rRNA	RRNA	0.68	0.0325	translation		
EIF3D	Eukaryotic translation initiation factor 3, subunit D	0.66	0.0018	Translation	0.008 ± 0.43	N.S.
EIF4G2	Eukaryotic translation initiation factor 4 gamma	0.66	0.0085	Translation	0.24 ± 0.19	N.S.
FAU	Finkel–Biskis–Reillymurine sarcoma virus (FBR-MuSV)	0.66	0.0310		0.66 ± 0.16	0.008
SART3	Squamous cell carcinoma antigen recognized by T cell 3	0.64	0.0140			
AC068228.1	tRNA pseudogene	0.63	0.0016			
RPL11	Ribosomal protein L11	0.62	0.0045			
C11orf58	Small acidic protein	0.61	0.0234			
C1D	Nuclear receptor co-repressor	0.6	0.0288			
HINT1	Adenosine 5′-monophosphoramidase	0.57	0.0212			
CBX3	Heterochromatin protein 1 homolog gamma	0.54	0.0129			
NPM1	Nucleophosmin 1	0.53	0.0090		0.25 ± 0.75	N.S.
RPS7	40S Ribosomal protein S7	0.52	0.0186			
HIST1H2BB	Histone H2B type 1-B	−0.57	0.0254			
SNORA42	Small nucleolar RNA	−0.65	0.0069			
RNU5E	RNA, U5E small nuclear	−0.86	0.0116			
AC025562.1	ScRNA pseudogene	−0.55	0.0210			
AL139097.1	RRNA pseudogene	−0.52	0.0086			
AL138832.1	RRNA pseudogene	−0.61	0.0023			
AP003461.5	Mt tRNA pseudogene	−0.51	0.0036			
AC105285.2	ScRNA pseudogene	−0.56	0.0297			
AL358813.3	tRNA pseudogene	−0.84	0.0064			
AL953889.1	ScRNA pseudogene	0.52	0.0356			
RP11613C6.4	Processed pseudogene	0.75	0.0390			
AL353774.1	tRNA pseudogene	0.55	0.0408			

^a^Data are expressed by mean of log2 fold change (FC). FC threshold cutoff ≤ ≥ 0.5

^b^Statistical significance of differences in microarray expression between supplemented PBMNC and baseline PBMNC, estimated by ANOVA test (*p* < 0.05)

^c^The overrepresented biological pathway was identified by DAVID functional annotation clustering tool

^d^Data are expressed as mean ± SD of log2 of fold change (FC)

^e^Statistical significance of differences in gene expression between supplemented PBMNC and baseline PBMNC, estimated by paired Student’s *t* test (*p* < 0.05; threshold cutoff ≤ ≥ 0.5)

^f^N.S. no significant difference

### Real-time PCR

A total of 1 μg of isolated RNA samples was reverse-transcribed into cDNA using the iScript™ Reverse Transcription Supermix (Bio-Rad) to confirm the expression of 6 genes upregulated in the Affymetrix assay. The quantification of gene expression was determined by real-time PCR (qPCR) with the 7500 Fast Real-Time PCR System (Applied Biosystem) using the iTaq™ Fast SYBR^®^ Green Supermix with ROX (Bio-Rad). Data were collected using the 7500 Software version 2.0.5 and given as threshold cycle (*C*_t_). C_t_ values for each target and reference genes were obtained, and their difference was calculated (Δ*C*_t_). Primer efficiencies for the test genes and the reference genes were similar. The comparative calculation, ΔΔ*C*_t_, was used to find the difference in the expression level between supplemented and baseline PBMNC. qPCR was also used to determine the expression of the cytokines, IL1A, IL1B, IL6, IL8, IL10, and TNFα in supplemented or baseline PBMNC, treated with LPS. Data are expressed as the mean of log2 of fold change (FC).

The oligonucleotides used for qPCR studies are as follows:

**Table:** 

Canx-(1)	FW: 5′TCCGCCTCTCTCTTTACTGC3′	RV: 5′CATGATCTCTAGCCTCCCGG3′
Canx-(2)	FW: 5′CGGCACGTGACGGTCGG3′	RV: 5′CCTTCCATGATCTTGCCCCGC3′
FAU	FW: 5′CTCCTGTTTGGCCACCTTAG3′	RV: 5′CTACCCTGGAAGTAGCAGGC3′
EIF3D	FW: 5′TCGCTGAGAGTCCAGCTTCT 3′	RV: 5′AACGGGGAAGTGTCCTTCAT3′
IK	FW: 5′ACGTGACTTAGAAGGTGGTGC3′	RV: 5′TGATCCTCACTCCTTCCACC3′
EIF4G2	FW: 5′CGGCTTGACAACGAAGAATC 3′	RV: 5′GGTGGCAGCTGCTGAGTT3′
NPM1	FW: 5′GGCGCTTTTTCTTCAGCTT3′	RV: 5′GGTCTGCCCCTGGAGGT3′
IL1A	FW: 5′CCGTGAGTTTCCCAGAAGAA3′	RV: 5′ACTGCCCAAGATGAAGACCA 3′
IL1B	FW 5′AAGCCCTTGCTGTAGTGGTG3′	RV: 5′GAAGCTGATGGCCCTAAACA3′
IL6	FW: 5′GTCAGGGGTGGTTATTGCAT3′	RV: 5′AGTGAGGAACAAGCCAGAGC 3′
IL8	FW: 5′AAATTTGGGGTGGAAGGTT3′	RV: 5′TCCTGATTTCTGCAGCTCTGT 3′
IL10	FW: 5′CTCATGGCTTTGTAGATGCCT3′	RV: 5′GCTGTCATCGATTTCTTCCC3′
TNFα	FW: 5′AGATGATCTGACTGCCTGGG3′	RV: 5′CTGCTGCACTTTGGAGTGAT3′
HPRT	FW: 5′TTAATGTAATCCAGCAGGCAGC3′	RV:5′CTCATGGACCTGATTATGGACAGG3′
βACTIN	FW: 5′AGAGCTAGCTGCCTGAC 3′	RV:5′GGATGCCACAGGACTCCA3′

### NF-κB-dependent gene expression

A total of 1 μg of RNA isolated from baseline or supplemented PBMNC, stimulated with LPS, was reverse-transcripted to analyze the expression of around 90 genes related to NF-κB pathway using the Human NFkB Signaling 96 StellARray™ qPCR array (Lonza) according to the manufacturer’s protocol. The quantification of gene expression was determined by qPCR as described before. Data are expressed as the mean of log2 of FC. Fold change (FC) threshold was set at ±1. The resulting differentially expressed genes were mapped to KEGG pathways in order to highlight specific pathways involved upon vitamin C treatment.

### Cytokines

Medium collected from PBMNC cultured with LPS was utilized to evaluate the expression and release of 12 different cytokines (IL1A, IL1B, IL2, IL4, IL6, IL8, IL10, IL12, IL17A, IFNγ, TNFα, and GM-CSF) by a Multi-Analyte ELISArray™ kit (SABiosciences) according to the manufacturer’s protocol.

### Statistical analysis

Microarray statistical analysis was performed utilizing OneChannel GUI R package (Sanges et al. 2007). Raw signal intensities were normalized utilizing GeneChip robust multiarray average (GCRMA) method employing the empirical Bayes approach for background correction followed by quantile normalization. Differentially expressed genes were identified using the “limma package,” applying linear models and moderated *t* statistics that implement empirical Bayes regularization of standard errors. Differences in expression values were expressed by mean of log2 of FC. A minimum difference threshold of FC ± 0.5 with a *p* value ≤0.05 was selected as gene differentially expressed in supplemented PBMNC with respect to baseline PBMNC.

Data obtained with vitamin C, qPCR, and ELISArray were analyzed using a paired Student’s *t* test.

The threshold for significance was set at *p* < 0.05 for all studies.

## Results

### Plasma total ascorbic acid

**Fig. 1:**
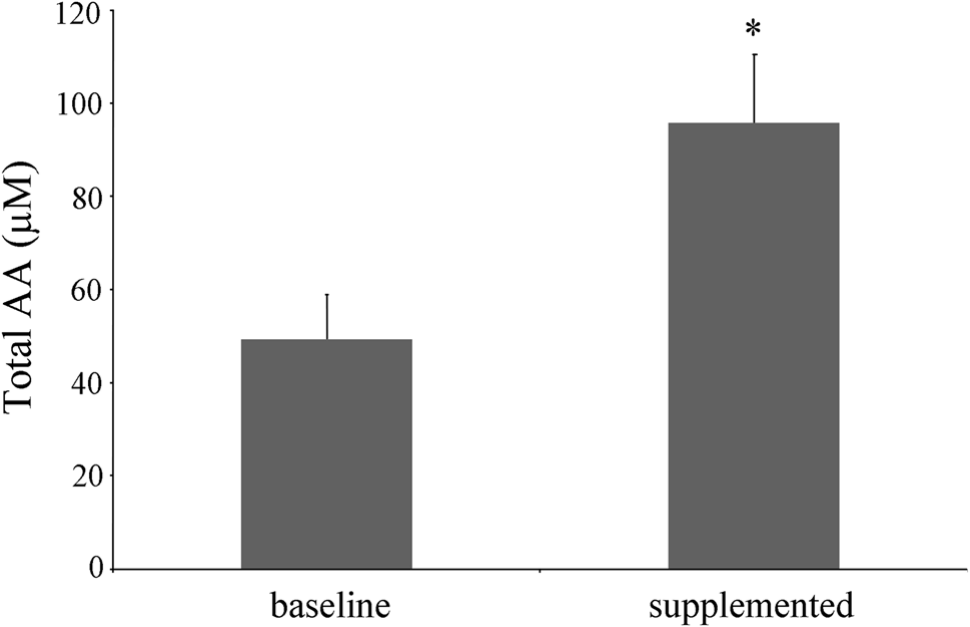
Plasma concentration of total ascorbic acid (AA) before and after the supplementation. *Significantly different *p* < 0.05 compared to baseline

Vitamin C is present in plasma and tissue mainly in its reduced state, i.e., ascorbate. However, it can be reversibly oxidized to DHA. Both forms have been measured in plasma, indicating that the majority of vitamin C was present in the reduced ascorbate form, while DHA was detectable in very small to negligible amounts (data not shown). As shown in Fig. 1, vitamin C administration almost doubled the mean plasma ascorbic acid concentration from the mean baseline concentration of 49.4 ± 9.6 to 95.8 ± 14 μM at the end of the 5 days of supplementation period.

### Effect of vitamin C on gene expression: microarray results

Microarray data indicate that 5 days of vitamin C supplementation induce a differential expression of only 1 gene, calnexin (CANX), which was upregulated by a FC value of 1.13 (*p* < 0.05, Table 1).

Usually, gene expression studies, assume a FC value higher than 1 as indicative of a *“bona fide”* biological difference. On the other hand, we can hypothesize that in the absence of deficiency conditions, an extra intake of a micronutrient normally consumed, only induces minute variations of gene expression on a quantitative scale, possibly resulting in a significant biological effect over a large window of time (Sanderson et al. 2008; Swali et al. 2011). We have therefore lowered the threshold to 0.5 log units, in order to characterize small quantitative changes, though statistically significant, that can be considered indicative of biological effects associated with vitamin C supplementation in healthy subjects. The adoption of this criteria lead to the identification of 39 genes modulated by vitamin C supplementation, including 9 pseudogenes characterized by a lack of functionality at protein level. The analysis of biological process (BP) enrichment indicates that 16 out of the 39 genes (around 40 %) mainly belong to BP related to ribonucleoprotein complex biosynthesis, translation, RNA processing, and chromatin organization pathway (Table 1).

### qPCR analysis

qPCR is the most sensitive and reproducible method to measure gene expression level. Several studies reported a good agreement between microarray and qPCR data, with correlation coefficients ranging from 0.69 to 0.80 (Dallas et al. 2005; Gyorffy et al. 2009). However, correlation coefficients decrease with decreasing in the degree of change of gene expression. Commonly, a single fold change (expressed as a difference in log_2_) is reported as the “cutoff” to a good correlation between microarray and qPCR data (Morey et al. 2006; Dallas et al. 2005).

As mentioned above, the setting of FC threshold at 0.5 allowed the identification of 39 genes modulated in vivo by AA. qPCR analysis was used to confirm the modulation of the expression of 6 genes (CANX, FAU, EIF3D, NPM1, IK, and EIF4G2) identified by microarray.

As shown in Table 1, a differential expression of only 2 genes, out of the subset of 6 genes analyzed, was confirmed to be statistically significant with a *p* value lower than 0.05 (FAU and CANX).

FAU is a gene that encodes for a fusion protein consisting of the ubiquitin-like protein *fubi* at the N terminus and ribosomal protein S30 at the C terminus (Pickard et al. 2011). Calnexin is a member of a family of molecular chaperones, assisting protein assembly and/or the retention within the endoplasmic reticulum of unassembled protein subunits (Wu et al. 2006). In particular, the FC expression of FAU (0.66) obtained by microarray was confirmed by qPCR; on the other hand, the differential expression value obtained for CANX gene by qPCR (0.53) was lower in comparison with the result obtained by microarray (1.13). Two transcript variants encoding for the same calnexin protein have been described (refseq code = NM_001746.3 CANX(1); NM_001024649.1 CANX(2)). Since gene array probesets do not allow to distinguish the expression of the two variants, we utilized two different sets of primers in order to discriminate the expression of the two variants by qPCR. After this technical refinement, we were able to identify a significant upregulation of the CANX(2) isoform, while the CANX(1) variant that represents the longest 4,953 bp transcript was not significantly modulated by vitamin C supplementation. These observations suggest that the total number of genes significantly modulated by AA is even lower than that provided by the microarray analysis and that vitamin C supplementation in healthy, well-nourished subjects is associated with a very modest effect on gene expression under normal physiological conditions.

### Vitamin C supplementation modulates gene expression inflammatory response in PBMNC treated with LPS

In order to assess the effect of vitamin C supplementation on cell response to an inflammatory stimulus, PBMNCs isolated before and after vitamin C supplementation were incubated in the presence of 10 % autologous plasma (containing 49.4 ± 9.6 and 95.8 ± 14 μM 0 ascorbate in baseline and supplemented plasma, respectively) with or without LPS for 5 h. As mentioned above, the presence of 10 % autologous plasma during the incubation of PBMNC with LPS was set in order to have a combinatory effect of the steady-state concentration of cellular vitamin C with the plasmatic AA concentration. At the end of the treatment, the expression of about 90 genes was simultaneously assessed by qPCR using a stellARray™ system. Setting a threshold ≤≥ ± 1 and *p* < 0.05, LPS treatment was found to be associated with a significant modulation of 22 genes, shared by PBMNC collected before and after Vitamin C supplementation (Table 2). On the other hand, seven genes were significantly upregulated by LPS only in PBMNC isolated before vitamin C supplementation (CFLAR, MAP2K3, MAP3K8, MYD88, TICAM1, TNF-α, and TRADD). Moreover, after AA supplementation, the upregulation of RELB gene was significantly inhibited by 1.43-fold. On the other hand, 6 genes were significantly downregulated by LPS only in PBMNC isolated after AA supplementation, (TNFRSF11A, MAP3K7IP1, MAPK14, PPP1R13L, TIRAP, and ZAP70) (Table 2). Most of the genes differentially regulated by LPS in PBMNC, isolated before and after AA supplementation, are involved in the toll-like receptor signaling pathway (hsa 04620) in agreement with the analysis performed with the Kegg analysis tool.

**Table 2:** Effect of LPS on gene expression level in PBMNC before and after vitamin C supplementation, analyzed using Human NFκB Signaling 96 STELLARRAY ™ qPCR array

Official gene symbol	Gene name	Baseline PBMNC	*p* value^a^	Supplemented PBMNC	*p* value^a^
		FC		FC	
BCL3	B-cell leukemia/lymphoma 3	2.08 ± 0.78	1 × 10^−3^	1.88 ± 0.51	5 × 10^−4^
EGR1	Early growth response 1	−3.01 ± 0.61	8 × 10^−4^	−3.99 ± 0.88	9 × 10^−6^
EIF2AK2	Eukaryotic translation initiation factor 2-alpha kinase 2	2.64 ± 0.89	3 × 10^−3^	2.84 ± 0.99	3 × 10^−4^
FOS	Proto-oncogene c-Fos	−4.52 ± 1.29	6 × 10^−4^	−5.88 ± 0.80	1.5 × 10^−7^
IRAK2	Interleukin-1 receptor-associated kinase 2	2.74 ± 1.04	3 × 10^−3^	2.77 ± 0.61	7 × 10^−4^
MAP2K6	Mitogen-activated protein kinase kinase 6	−3.33 ± 1.16	1 × 10^−3^	−3.36 ± 1.18	1 × 10^−4^
MAP3K3	Mitogen-activated protein kinase kinase kinase 3	−1.35 ± 0.76	1 × 10^−2^	−1.64 ± 0.57	5 × 10^−4^
MAPK11	Mitogen-activated protein kinase 11	2.25 ± 0.58	6 × 10^−4^	2.40 ± 0.45	2 × 10^−5^
MAPK3	Mitogen-activated protein kinase 3	−1.14 ± 0.46	3 × 10^−2^	−1.81 ± 0.41	6 × 10^−3^
NFKB1	Nuclear factor NF-kappa-B p105 subunit	2.64 ± 0.43	1 × 10^−4^	2.36 ± 0.17	3 × 10^−6^
NFKB1A	NF-kappa-B inhibitor alpha	1.82 ± 0.62	6 × 10^−3^	1.07 ± 0.88	3 × 10^−2^
NFKB2	Nuclear factor NF-kappa-B p100 subunit	2.33 ± 0.18	1 × 10^−5^	2.02 ± 0.52	9 × 10^−4^
STAT1	Signal transducer and activator of transcription 1-alpha/beta	3.69 ± 0.86	4 × 10^−5^	3.82 ± 0.18	4 × 10^−8^
TIFA	TRAF-interacting protein with forkhead-associated domain	1.97 ± 0.60	1 × 10^−3^	1.84 ± 0.53	1 × 10^−5^
TNFAIP3	Tumor necrosis factor, alpha-induced protein 3	1.43 ± 0.55	4 × 10^−3^	1.30 ± 0.50	1 × 10^−3^
TNIP2	TNFAIP3-interacting protein 2	1.05 ± 0.52	1 × 10^−2^	1.10 ± 0.35	8 × 10^−3^
TRAF3IP2	Adapter protein CIKS	2.04 ± 1.06	1 × 10^−3^	2.25 ± 0.57	2 × 10^−3^
IL1A	Interleukin-1 alpha	6.10 ± 2.83	8 × 10^−3^	5.84 ± 1.68	1 × 10^−3^
IL1B	Interleukin-1 beta	3.47 ± 1.61	8 × 10^−3^	3.16 ± 1.07	3 × 10^−3^
IL6	Interleukin-6	9.24 ± 2.36	9 × 10^−4^	10.02 ± 1.48	1 × 10^−4^
IL8	Interleukin-8	2.66 ± 1.70	2 × 10^−2^	2.43 ± 1.33	1 × 10^−2^
IL10	Interleukin-10	3.30 ± 1.42	3 × 10^−3^	3.02 ± 0.96	1 × 10^−3^
CFLAR	CASP8 and FADD-like apoptosis regulator precursor	1.05 ± 0.4	6 × 10^−3^	0.92 ± 0.34	N.S^b^
MAP2K3	Mitogen-activated protein kinase kinase 3	1.83 ± 0.81	2 × 10^−3^	0.97 ± 1.41	N.S.
MAP3K7IP1	Mitogen-activated protein kinase kinase kinase 7-interacting protein 1	0.017 ± 0.60	N.S.	−1.04 ± 0.45	1 × 10^−2^
MAP3K8	Mitogen-activated protein kinase kinase kinase 8	2.89 ± 0.88	2 × 10^−3^	2.1 ± 2.27	N.S.
MAPK14	Mitogen-activated protein kinase 14	−0.82 ± 0.94	N.S.	−1.19 ± 0.71	3 × 10^−2^
MYD88	Myeloid differentiation primary response protein MyD88	1.12 ± 0.40	6 × 10^−3^	0.5 ± 0.46	N.S.
PPP1R13L	RelA-associated inhibitor	−0.49 ± 0.87	N.S.	−1.18 ± 0.66	2 × 10^−2^
RELB	Transcription factor RelB	2.68 ± 1.22	5 × 10^−3^	1.24 ± 0.53	7 × 10^−4^
TICAM1	Toll-like receptor adaptor molecule 1	1.37 ± 0.59	2 × 10^−2^	0.94 ± 0.61	N.S.
TIRAP	Toll-interleukin 1 receptor (TIR) domain containing adaptor protein	−0.47 ± 0.21	N.S.	−1.00 ± 0.41	7 × 10^−3^
TNFRSF11A	Tumor necrosis factor receptor superfamily member 11A precursor	−1.32 ± 1.13	N.S.	−3.24 ± 0.82	3 × 10^−4^
TRADD	Tumor necrosis factor receptor type 1-associated DEATH domain protein	1.51 ± 0.67	1 × 10^−2^	0.89 ± 0.32	N.S.
TNF-α	Tumor necrosis factor-alpha	2.75 ± 0.83	2 × 10^−3^	1.26 ± 1.55	N.S.
ZAP70	Tyrosine-protein kinase ZAP-70	−0.23 ± 0.26	N.S.	−1.11 ± 0.45	8 × 10^−3^

Data are expressed by mean ± SD of log2 fold change (FC) (FC threshold cutoff ≤≥1)

^a^Statistical significance of differences in gene expression between LPS treated and control both in baseline and supplemented PBMNC, estimated by paired Student’s *t* test (*p* < 0.05)

^b^N.S. no significant difference

### Effect of vitamin C supplementation on cytokine release in PBMNC challenged with LPS

**Fig. 2:**
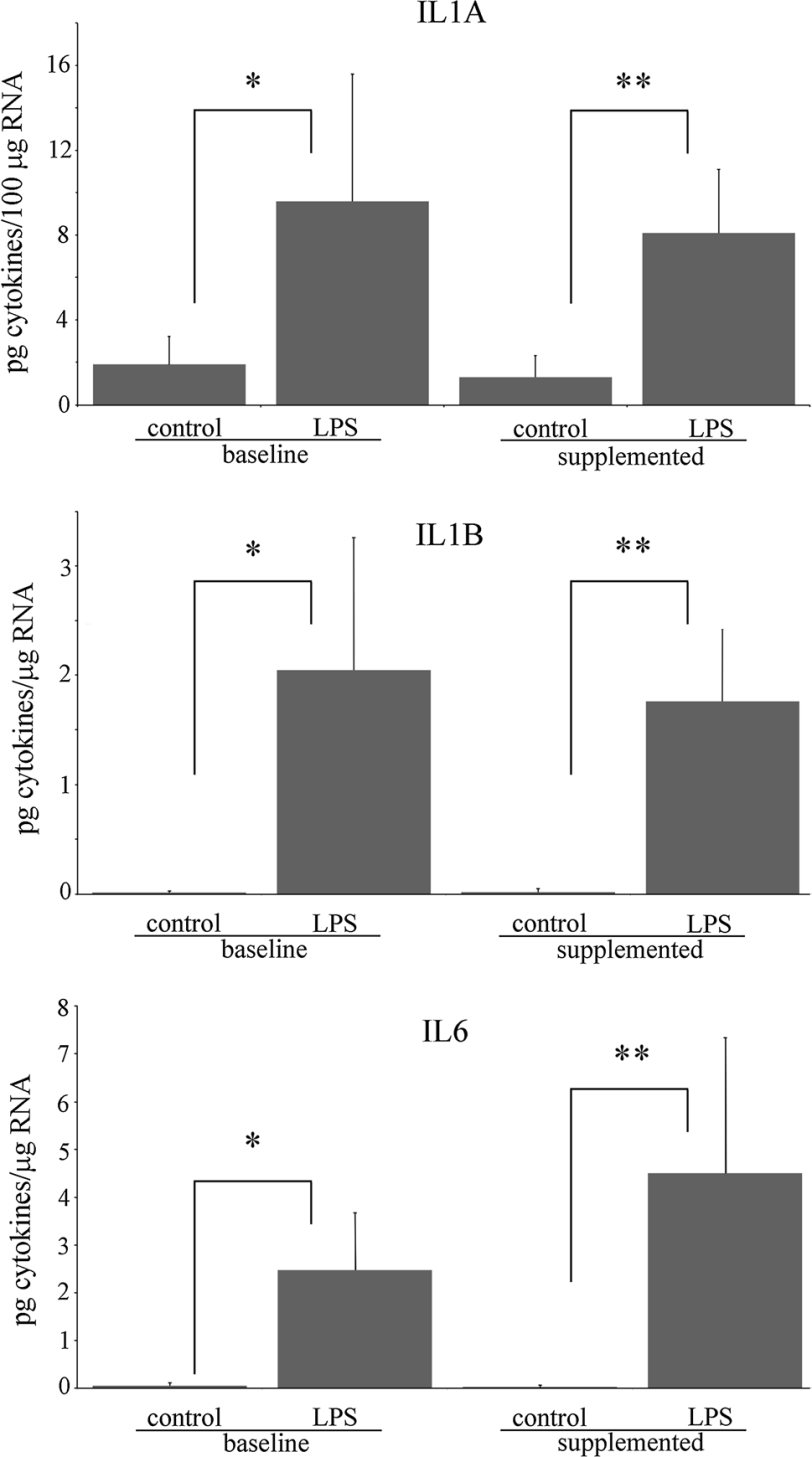
Effect of LPS on IL1A, IL1B, and IL6 release in PBMNC isolated before and after vitamin C supplementation. *Significantly different, *p* < 0.05, compared to control in baseline PBMNC; **Significantly different, *p* < 0.05, compared to control in supplemented PBMNC

**Fig. 3:**
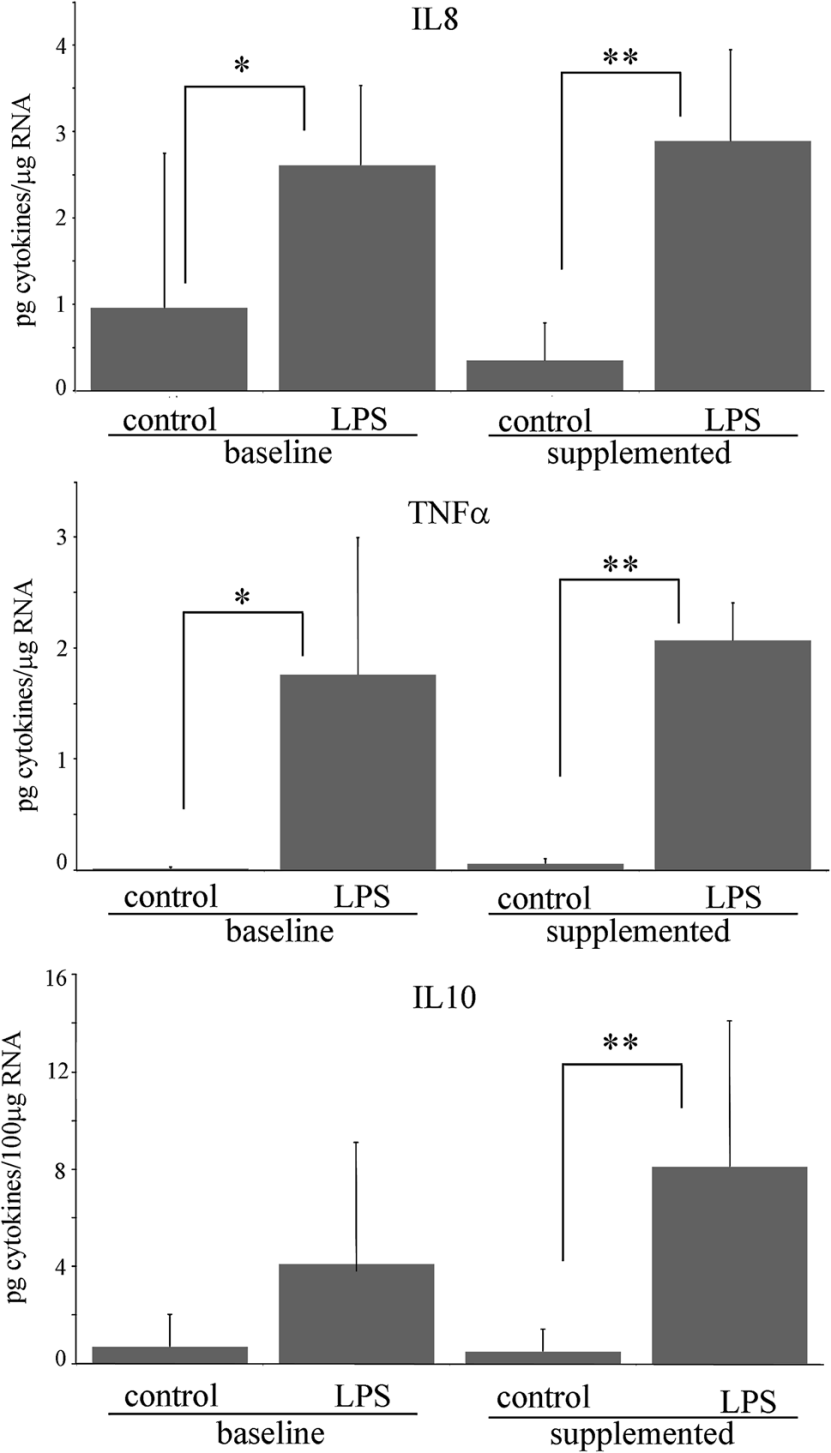
Effect of LPS on IL8, TNFα, and IL10 release in PBMNC isolated before and after vitamin C supplementation. *Significantly different, *p* < 0.05, compared to control in baseline PBMNC; **Significantly different, *p* < 0.05, compared to control in supplemented PBMNC

In order to study the effect of vitamin C supplementation on cytokine release induced by LPS, the release of 12 different cytokines was measured in the medium collected at the end of the incubation. Our results indicate that LPS activates the synthesis and release of the cytokines IL1A, IL1B, IL6, IL-8, and TNF-α, both in PBMNC isolated before and after vitamin C supplementation (Figs. 2, 3). A significant release of cytokine IL-10 in comparison with control was associated with LPS treatment only in PBMNC isolated after AA supplementation (Fig. 3). On the other hand, cytokines IL2, IL4, IL12, IL17A, and GM-CSF were not released in the medium at the end of the incubation with LPS both in supplemented and baseline PBMNC.

## Discussion

A significant body of evidence suggests that there is a positive association between vitamin C status and health. The dietary intake recommendation (RDA) for vitamin C has been set by scientific bodies and health authorities worldwide with the intention of preventing vitamin C deficiency and the associated disease, scurvy (Frei et al. 2012). The revised European RDA for vitamin C is 80 mg/day (European Commission 2008). Vitamin C absorption is tightly controlled. In fact, plasma concentration unlikely exceeds 100 μM, after massive oral consumption (Levine et al. 1996). Concentration of serum AA higher than 28 μM is considered to be adequate, while amount lower than 11 μM would indicate vitamin C deficiency (Jacob 1990). Irrespective of the amount of vitamin C consumed, plasmatic ascorbate concentration is usually low in smokers (Alberg 2002) and in patients with pancreatitis, acute myocardial infarction, and diabetes (Bonham et al. 1999) (Riemersma et al. 2000) (Price et al. 2001). Moreover, nonfunctional genotypes of the glutathione S-transferase enzyme (GSTT1 and/or GSTM1) are associated with a higher risk of serum vitamin C deficiency (Cahill et al. 2009). In our study, the mean baseline concentration of AA measured in the healthy volunteers was 49 μM, indicating an adequate “vitamin C status.” A dietary intake of 60 mg/day vitamin C has been shown to correspond to a median plasma concentration around 40 μM in healthy subjects (Levine et al. 1996). On the other hand, at the end of the supplementation, plasmatic AA increased in all the subjects up to about 95.8 μM.

The metabolic and biochemical properties of AA have been extensively documented. Most of them are directly associated with the chemical nature and reducing activity of the molecule. While some vitamins (such as vitamins A, D, or E) have been demonstrated to act as signaling molecules, the role of AA in gene expression modulation is still largely unknown. Ascorbic acid (AA) has been shown to regulate the expression of genes encoding extracellular matrix proteins and to stimulate procollagen mRNA at transcriptional level (Ronziere et al. 2003; Chojkier et al. 1989). Moreover, AA downregulates the expression of genes necessary for S-phase progression and induces necrotic cell death in primary cultured fibroblasts (Belin et al. 2009). So far, no data are available on the effect of AA supplementation on gene modulation in circulating leukocytes. In the present study, we made a broad spectrum analysis to detect the effects of a supplementation of vitamin C on circulating WBC at molecular level, utilizing a microarray technology.

In our study, vitamin C supplementation had a very small effect on gene expression profile in PBMNC. Even considering a FC ≤≥ 0.5, rather than a most utilized threshold ≤≥ 1, only few genes resulted to be modulated in all the 5 subjects recruited, mainly involved into the BP related to ribonucleoprotein complex biosynthesis, translation, RNA processing, and chromatin organization pathway. Moreover, data obtained by gene array were not fully confirmed by qPCR analysis. This discrepancy supports the limits of sensitivity of microarray technology as compared to qPCR and confirms the low degree of changes induced by AA on PBMNC under normal physiological conditions. The limited entity of differences in observed FC, the small number of genes affected by vitamin C supplementation, and low the power value (0.07) calculated for the microarray study suggest that in physiological condition, the few differently expressed genes observed after supplementation have a very small (if any) biological relevance.

In order to better identify a specific cellular response associated with vitamin C supplementation, PBMNC obtained before and after vitamin C supplementation were stimulated with a pro-inflammatory second “hit” constituted of an exposure to LPS. It is known that LPS induces a strong response in immune system, and acting as an endotoxin, it binds the CD14/TLR4/MD2 receptor complex, promoting the secretion of pro-inflammatory cytokines in different cell types (Chow et al. 1999). The role of vitamin C in immune function has been widely studied in the last 3 decades (Wintergerst et al. 2006) (Webb and Villamor 2007) (Jacob et al. 1991). Beside its antioxidant properties and its role in collagen synthesis, vitamin C has been shown to improve phagocytic function and has an immunomodulatory effect on lymphocyte cells (Anderson et al. 1980). It also has been shown that large doses of vitamin C might markedly lower blood histamine concentrations, which is inversely associated with leukocyte chemotaxis (Johnston et al. 1994). Vitamin C deficiency is associated with a decreased resistance to diseases and some evidence suggests that it might have antiviral activity in humans (Mortola et al. 1998) (Jariwalla and Harakeh 1998). In fact, vitamin C is frequently used in the treatment and prevention of common cold (Heimer et al. 2009). A recent meta-analysis of well-conducted clinical studies finally concluded that vitamin C administration provides a significant benefit on duration and severity of common cold (Douglas et al. 2007).

Vitamin C has been shown to play a significant role in the regulation of inflammatory response, but its effect on inflammatory response is controversial. It has been reported (Wannamethee et al. 2006) that vitamin C status and the consumption of vitamin C-rich food items (fruits and vegetables) is associated with lower inflammatory biomarkers in men with no history of cardiovascular disease or diabetes. Oral vitamin C has also been reported to downregulate some proinflammatory mediators in smokers bearing apoE4 allelic variant (Majewicz et al. 2005). On the other hand, other studies were not able to observe any association between vitamin C supplementation and the secretion of inflammatory cytokines, ox-LDL (Lu et al. 2005), or on the oxidative/antioxidative stress and inflammatory markers in hemodialysis patients (Fumeron et al. 2005). Similarly, other reports indicate that vitamin C has no effect on TNF and prostaglandin E2 production induced by LPS (Jeng et al. 1996). Previous studies have shown that vitamin C has an immunomodulatory activity by inhibiting the LPS-induced number of monocytes producing IL-6 and TNF-alpha (Hartel et al. 2004).

**Fig. 4:**
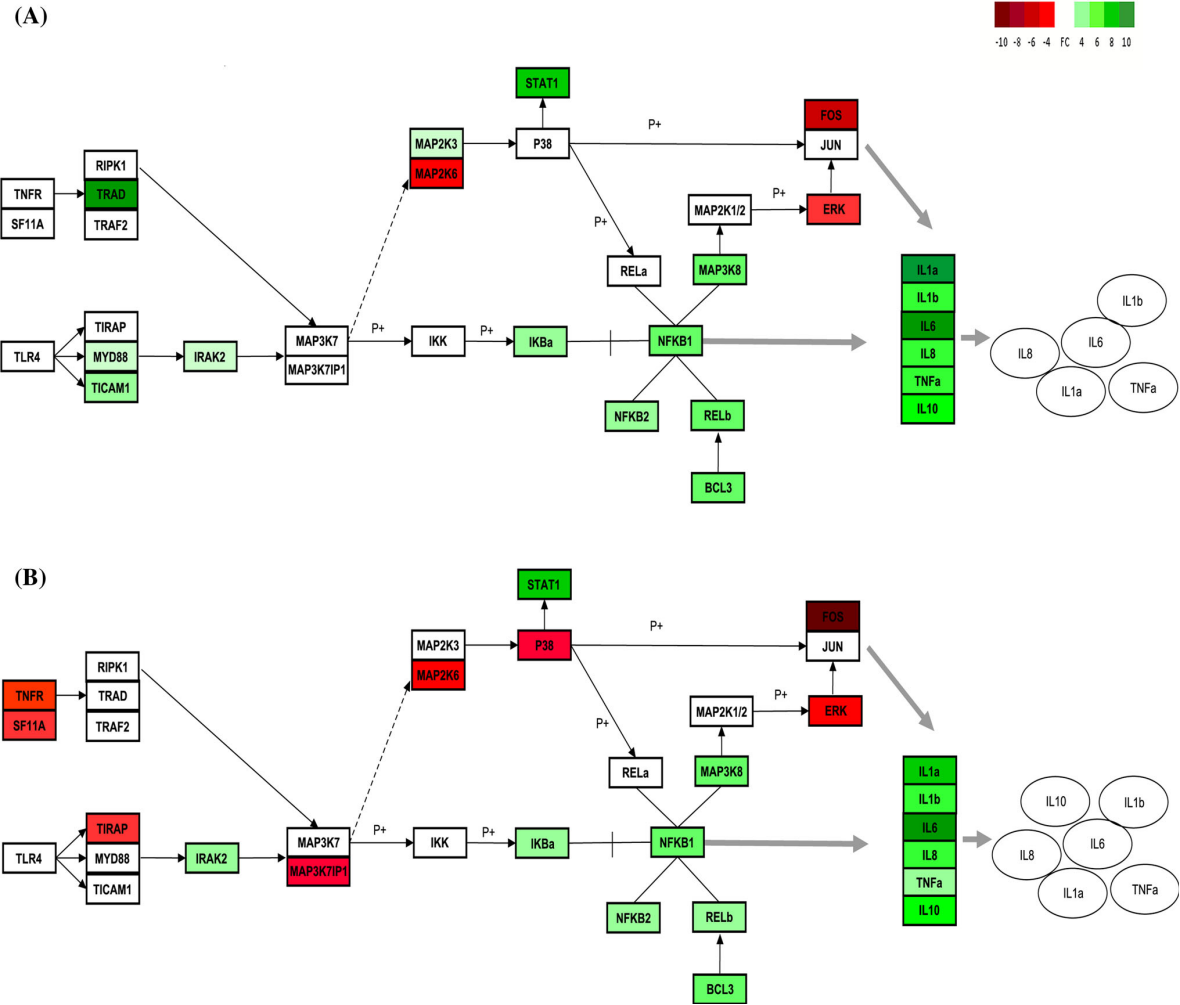
Description of LPS-dependent gene expression modulation within MyD88-dependent pathway in PBMNC. **a** Before the supplementation, **b** after the supplementation. Rectangles represent FC of gene expression, and circles represent the cytokines release in the medium at the end of LPS incubation

Our data showed that after 5 h of incubation with LPS, the expression of several genes involved in toll-like receptor pathway, in particular the MYD88-dependent pathway (Fig. 4, panel a), is induced in PBMNC isolated before AA supplementation. This event is in turn associated with the activation of NF-κB transcription factor and MAPK signaling cascades, finally resulting in the increase in the expression of mRNA encoding for TNF-α, IL-1B, IL1A, IL6, and IL8 (Underhill and Ozinsky 2002; Chow et al. 1999). The treatment of PBMNC with LPS also induced the release of the cytokines, IL1A, IL1B, IL6, IL8, and TNF-α in the medium, indicating that mRNA transcription that anticipates the synthesis and release of these proteins was already active during the first stages of the pro-inflammatory treatment.

LPS activation of PBMNC isolated after vitamin C supplementation was associated with an evident decrease in the early steps of Myd88 pathway and, ultimately, to the decrease in TNF-α mRNA transcription (Fig. 4, panel b). In fact, the expression of MYD88, TICAM1, and MAP2K3 and TNFα levels were comparable to those observed in unstimulated cells (Table 2). Moreover, vitamin C supplementation was associated with a downregulation of TIRAP, P38 and MAP3K7IP1, and to a decreased activation of RELB.

The pattern of cytokine release associated with LPS treatment was similar before and after the supplementation, with the exception of IL-10 that was not significantly detectable in the medium of PBMNC isolated before the supplementation. IL10 is considered an anti-inflammatory cytokine by virtue of its inhibitory activity on cytokine synthesis. In fact, IL-10 synthesis is a relatively late event in comparison with other monokines (Wang et al. 1994). Once synthesized and released, this cytokine can limit or terminate the inflammatory responses by inhibiting pro-inflammatory cytokine production either as mRNA accumulation or protein release (Wang et al. 1994; de Waal et al. 1991; Rajasingh et al. 2006), allowing an appropriate balance between pro- and anti-inflammatory effectors. Moreover, one of the proposed molecular mechanisms associated with IL-10 anti-inflammatory activity is the regulation of MYD88-dependent pathway (Chang et al. 2009). Our results indicate that at 5 h from LPS treatment, IL-10 is already present in the culture medium of the PBMNC isolated after vitamin C supplementation. Our data also suggest the presence of an early activation of IL-10 synthesis associated with the presence of vitamin C that could result in a different modulation of genes involved in MYD88-dependent pathway (Fig. 4).

As already mentioned in the introduction, this study has an “exploratory” nature, aimed to provide a conceptual background for a further exploitation of the understanding of the mechanisms of the molecular effects of AA. In fact, in spite of the limited number of subjects analyzed, a significant specific modulation of cell response to inflammation has been observed.

In conclusion, our study suggests that vitamin C supplementation in healthy subjects, not selected according to a specific genetic profile predisposing to high vitamin needs, consuming an adequate vitamin C amount, and therefore having a satisfactory vitamin C plasma concentration, is not associated with a significant change in gene expression profile. This is possibly also true for a number of bioactive molecules either belonging to the family of established micronutrients (vitamins and minerals) or to the less characterized family of the so-called bio-active molecules (e.g., polyphenols). Under this “satisfactory nutritional status,” also important supplementations (such as 1 g vitamin C supplementation) are not immediately evident and are “buffered” within a homeostatic physiological equilibrium. Differently, following a second “hit” such as the one considered in the ex vivo part of our study constituted of the inflammatory stimulus LPS, able to trigger a critical burst to the normal physiological state, the higher availability of ascorbic acid emerges, and results in a significant modulation of cell response (Muller and Kersten 2003).

## Abbreviations

AA: Ascorbic acid
BP: Biological process
MPA: Metaphosphoric acid
PBMNC: Peripheral blood mononuclear cells
LPS: Lipopolysaccharides
DHA: Dehydroascorbic acid
RBC: Red blood cell
WBC: White blood cell
GCRMA: GeneChip robust multiarray average
GO: Gene ontology
FC: Fold change
